# Uncovering the impact of alcohol on internal organs and reproductive health: Exploring *TLR4/NF‐kB* and CYP2E1/ROS/Nrf2 pathways

**DOI:** 10.1002/ame2.12436

**Published:** 2024-06-09

**Authors:** Eason Qi Zheng Kong, Vetriselvan Subramaniyan, Natasha Sura Anak Lubau

**Affiliations:** ^1^ Pharmacology Unit, Jeffrey Cheah School of Medicine and Health Sciences Monash University Malaysia Subang Jaya Selangor Malaysia; ^2^ Center for Global Health Research, Saveetha Medical College Saveetha Institute of Medical and Technical Sciences Chennai Tamil Nadu India

**Keywords:** alcohol, health impact, inflammation, metabolism, molecular pathways

## Abstract

This review delves into the detrimental impact of alcohol consumption on internal organs and reproductive health, elucidating the underlying mechanisms involving the *Toll‐like receptor 4 (TLR4)*/Nuclear factor kappa light chain enhancer of activated B cells (NF‐kB) pathway and the Cytochrome P450 2E1 (CYP2E1)/reactive oxygen species (ROS)/nuclear factor erythroid 2‐related factor 2 (Nrf2) pathways. The *TLR4/NF‐kB* pathway, crucial for inflammatory and immune responses, triggers the production of pro‐inflammatory agents and type‐1 interferon, disrupting the balance between inflammatory and antioxidant responses when tissues are chronically exposed to alcohol. Alcohol‐induced dysbiosis in gut microbes heightens gut wall permeability to pathogen‐associated molecular patterns (PAMPs), leading to liver cell infection and subsequent inflammation. Concurrently, CYP2E1‐mediated alcohol metabolism generates ROS, causing oxidative stress and damaging cells, lipids, proteins, and deoxyribonucleic acid (DNA). To counteract this inflammatory imbalance, Nrf2 regulates gene expression, inhibiting inflammatory progression and promoting antioxidant responses. Excessive alcohol intake results in elevated liver enzymes (ADH, CYP2E1, and catalase), ROS, NADH, acetaldehyde, and acetate, leading to damage in vital organs such as the heart, brain, and lungs. Moreover, alcohol negatively affects reproductive health by inhibiting the hypothalamic–pituitary‐gonadal axis, causing infertility in both men and women. These findings underscore the profound health concerns associated with alcohol‐induced damage, emphasizing the need for public awareness regarding the intricate interplay between immune responses and the multi‐organ impacts of alcohol consumption.

## Graphical abstract

Chronic alcohol exposure disrupts *toll‐like receptor 4/*nuclear factor kappa light chain enhancer of activated B cells and Cytochrome P450 2E1/reactive oxygen species/nuclear factor erythroid 2‐related factor 2 pathways, causing inflammation, gut dysbiosis, liver issues, and oxidative stress in vital organs. Excessive alcohol intake inhibits the hypothalamic–pituitary‐gonadal axis, negatively impacting fertility. This highlights the urgent need for public awareness of the intricate interplay between immune responses and the widespread health effects of alcohol consumption.

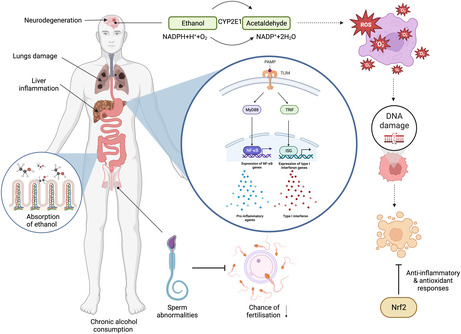

## 1 INTRODUCTION

Alcohol, a globally prevalent habit, exerts profound health consequences that extend beyond its well‐documented impact on the liver. This review focuses on the intricate interplay between the *toll‐like receptor 4* (*TLR4*)/nuclear factor kappa light chain enhancer of activated B cells (NF‐kB) pathway and the cytochrome P450 2E1 (CYP2E1)/reactive oxygen species (ROS)/nuclear factor erythroid 2‐related factor 2 (Nrf2) pathway. These molecular events underlie the detrimental effects of chronic alcohol intake on internal organs and reproductive health, disrupting the delicate balance between inflammatory and antioxidant responses.^1^, ^2^, ^3^

The *TLR4/NF‐kB* pathway, crucial for inflammatory and immune responses, recognizes pathogen‐associated molecular patterns (PAMPs) in microorganisms.^4^, ^5^ Chronic alcohol consumption disrupts gut microbial equilibrium, increasing permeability to PAMPs and causing inflammation in internal organs like the heart, brain, and lungs.^2^, ^6^ The CYP2E1/ROS/Nrf2 pathway, initiated by alcohol metabolism, leads to oxidative stress and cellular damage. Nrf2 acts as a countermeasure, regulating gene expression to restore the disrupted balance.^7^ This review also explores alcohol's negative impact on reproductive health, inhibiting the hypothalamic–pituitary‐gonadal (HPG) axis and causing infertility.^8^, ^9^ Exploring the *TLR4* signal pathway, we examine its structure, activation mechanisms, and downstream pathways, emphasizing their roles in alcohol‐induced damage. In summary, this exploration highlights the complex nature of immune responses and the urgent need for public awareness regarding the widespread health consequences of excessive alcohol consumption.

## 2 *TLR4* SIGNAL PATHWAY: UNVEILING THE INTRICACIES OF HOST DEFENSE

**FIGURE 1:**
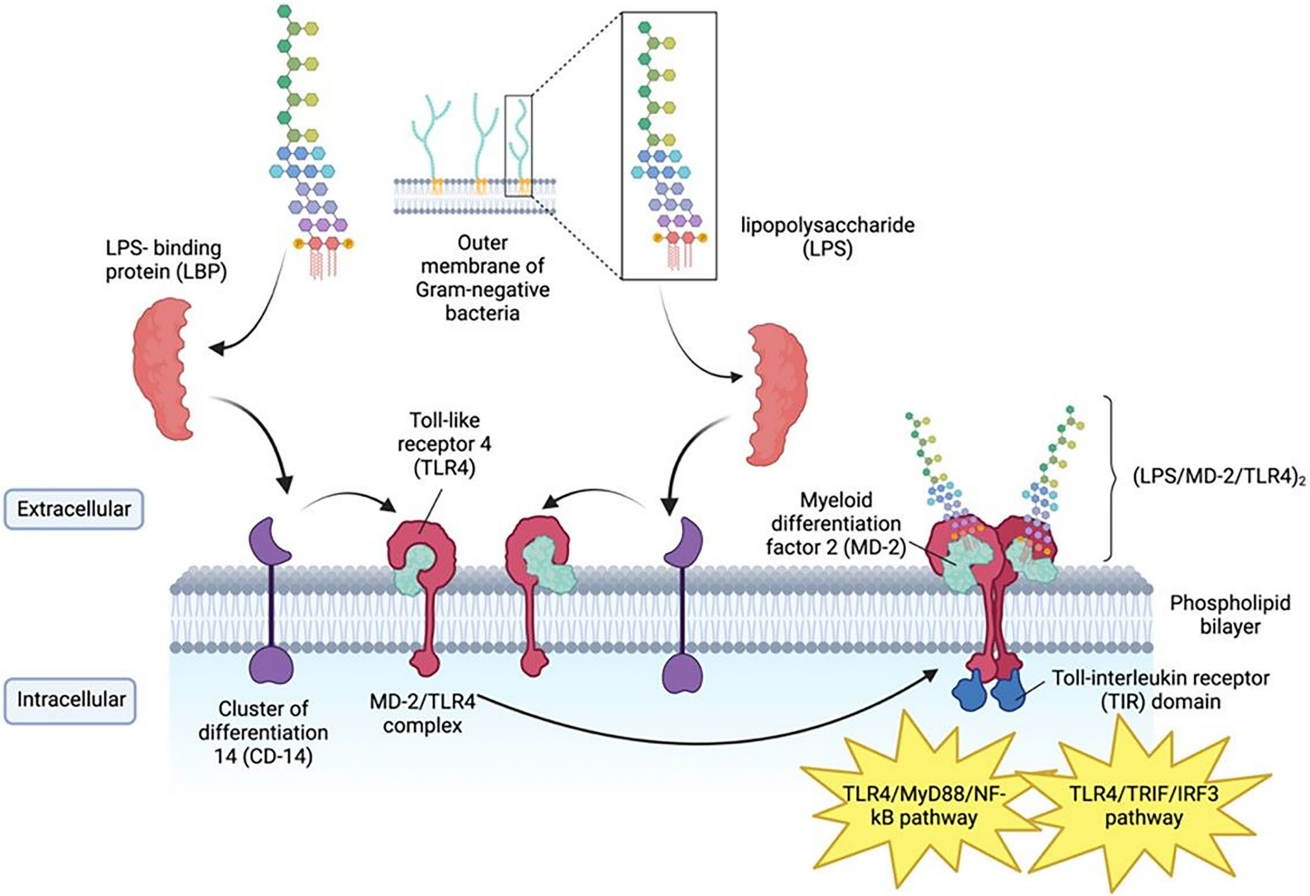
*Toll‐like receptor 4* (*TLR4*) signal pathway, highlighting key molecular interactions and signaling events involved in the recognition of lipopolysaccharide (LPS) and subsequent activation of downstream pathways.

Excessive alcohol consumption increases the intestinal level of miR‐212 that binds to the tight junction of epithelial cells, namely zona occludens‐1 (ZO‐1), inhibiting the synthesis of its mRNA. This increases the gut permeability to PAMPs such as fungi, bacteria, parasites and viruses, enabling the translocation of bacteria from intraintestinal lumen into extraintestinal space, transporting alcohol molecule to the blood stream, and activating the *TLR4* signal pathway.^10^, ^11^ *TLR4* is a transmembrane protein that recognizes lipopolysaccharide (LPS), one of the components found in the outer membrane of Gram‐negative bacteria, and it stimulates the immune response against these bacteria.^12^, ^13^, ^14^, ^15^ In the membrane, *TLR4* monomers are associated with adaptor protein myeloid differentiation factor 2 (MD‐2), and cluster of differentiation 14 (CD‐14).^16^, ^17^, ^18^ In the extracellular fluid, LPS is bound by LPS‐binding protein (LBP), then transferred to CD‐14, which allows interaction with the MD‐2/*TLR4* complex.^13^, ^14^ The dimerization of *TLR4* occurs after formation of the final activated heterodimer (LPS/MD/*TLR4*)_2_ on the extracellular side, and interaction between two toll‐interleukin receptor (TIR) domains and the two *TLR4* monomers on the intracellular side.^19^, ^20^ This process activates the intracellular signals, which are the myeloid differentiation primary response 88 (My‐D88) dependent pathway and the TIR‐domain‐containing adapter‐inducing interferon‐β (TRIF) dependent pathway.^21^, ^22^ The MyD88, TIR domain, toll interleukin 1 receptor adaptor protein (TIRAP), TRIF‐related adaptor molecule (TRAM) and TRIF are necessary for both pathways to occur^23^ (Figure 1).

Chronic alcohol consumption activates the *TLR4*/*NF‐κB* pathway, inducing a cascade of pro‐inflammatory responses that contribute to tissue damage in internal organs such as the liver. Activation of NF‐κB triggers the release of inflammatory mediators and cytokines, promoting inflammation and tissue injury over time. This mechanism highlights the intricate interplay between alcohol‐induced inflammation and the development of organ damage.

## 3 MY‐D88 DEPENDENT PATHWAY: UNRAVELING THE ROLE OF PRO‐INFLAMMATORY CYTOKINES

**FIGURE 2:**
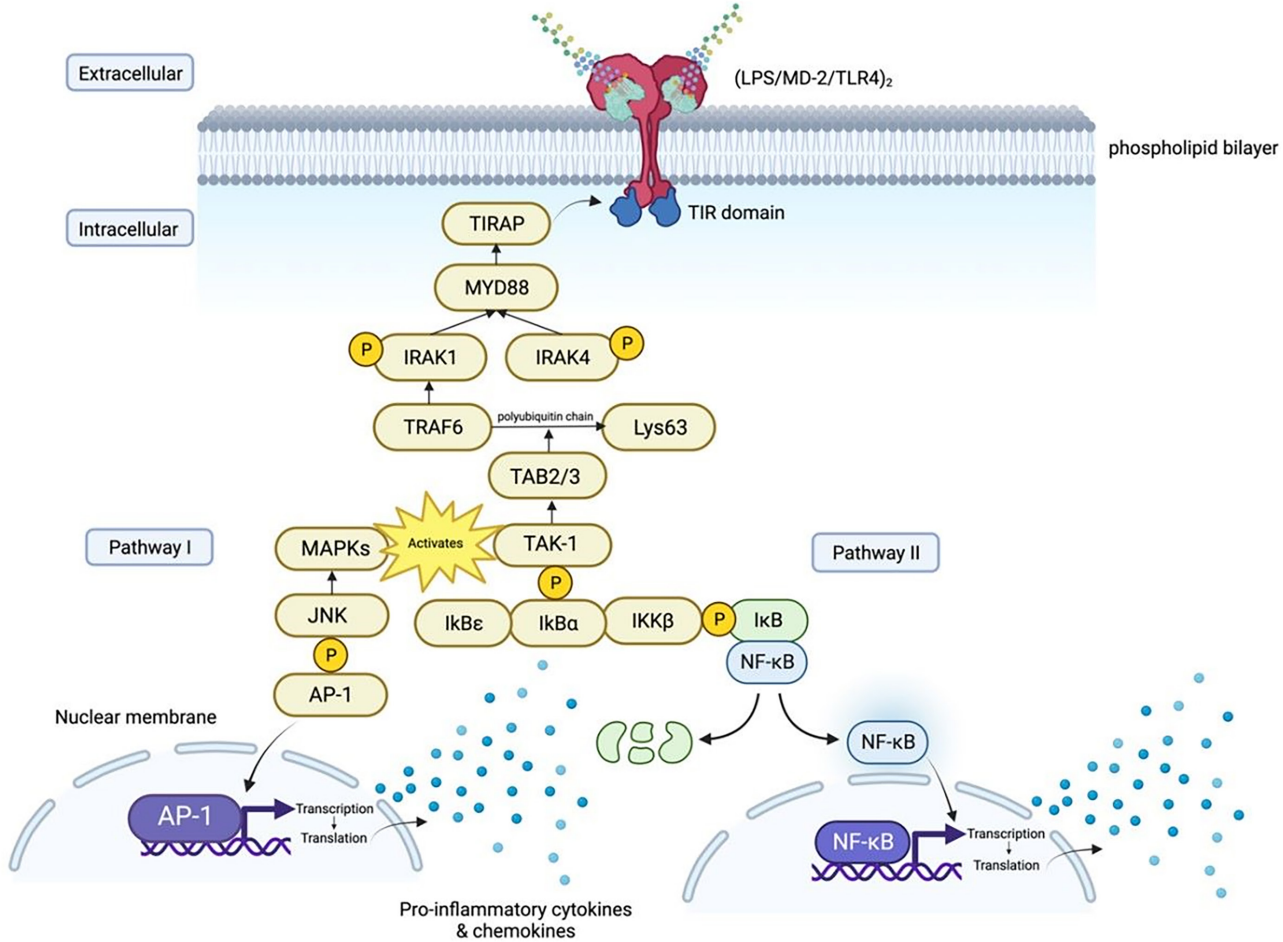
The figure depicts the MyD88‐dependent pathway, elucidating the molecular events from *TLR4* activation to the transcription of pro‐inflammatory cytokines. Key interactions involving TIRAP, MyD88, IRAKs, TRAF6, TAK‐1, and downstream signaling components. IRAK, interlukin‐1 receptor‐associated kinases; TIRAP, toll interleukin 1 receptor adaptor protein; TLR4, toll‐like receptor 4; TRAF6, TNF receptor associated factor 6.

As shown in Figure 2, In the MyD88‐dependent pathway, the TIR‐TIR dimer recruits TIRAP, which contains a phosphatidylinositol 4,5‐biphosphate‐binding domain, serving as a site to join with MyD88.^19^, ^24^ MyD88 binds to interlukin‐1 receptor‐associated kinases 2 (IRAK 2) and interlukin‐1 receptor‐associated kinases 4 (IRAK4), forming myddosome.^20^, ^25^ This process leads to autophosphorylation of IRAK4 and phosphorylation of IRAK1 by IRAK4.^23^, ^26^ TNF receptor associated factor 6 (TRAF6) forms a trimer with phosphorylated IRAK1, and attaches itself to the amino acid lysine at position 63 (Lys63) by polyubiquitin chains, which combine with adaptor protein TAK‐1 binding protein 2/TAK‐1 binding protein 3 (TAB2/TAB3).^27^ This complex activates TGF‐beta activated kinase (TAK‐1) protein, which phosphorylates the inhibitor of the nuclear factor kB (IkB) kinase (IKK) complex, thereby activating it to unmask the nuclear localization of NF‐kB by NF‐kB inhibitor alpha (IkBα).^22^, ^26^, ^27^ NF‐kB enters the nucleus and drives the transcription of genes that produce pro‐inflammatory cytokines and chemokines.^16^, ^23^ Furthermore, there is another signaling branch coming from TAK‐1 protein which activates the mitogen‐activated protein kinases (MAPKs). The MAPKs recruit c‐Jun NH2‐terminal kinase (JNK) protein, which phosphorylates the activating protein‐1 (AP‐1) transcription factor to transcribe pro‐inflammatory cytokines and chemokines, resulting in recruitment of inflammatory cells.^28^

## 4 TRIF‐DEPENDENT PATHWAY: UNVEILING THE INFLUENCE ON TYPE‐1 INTERFERONS

In the TRIF‐dependent pathway, TRIF‐related adaptor molecule (TRAM) protein, which is a membrane‐bound bridging adaptor, is recruited to TIR domain.^20^, ^22^, ^25^ It recruits and activates TRIF, followed by activation of receptor‐interacting serine/threonine‐protein kinase 1 (RIP‐1), TNF receptor associated factor 3 (TRAF‐3), TANK‐binding kinase 1 (TBK‐1), and interferon regulatory factor 3 (IRF‐3) proteins, which will be phosphorylated and transferred into the nucleus to initiate transcription of type‐1 interferon gene.^29^

Type‐1 IFN binds to a heterodimeric transmembrane receptor that contains subunits of interferon alpha and beta receptor subunit 1 (IFNAR1) and interferon alpha and beta receptor subunit 2 (IFNAR2). Ligation of IFNAR activates the receptor‐associated protein tyrosine kinases Janus kinase 1 (JAK1), tyrosine kinase 2 (TYK2), signal transducer and activator of transcription 1 (STAT1) and signal transducer and activator of transcription 2 (STAT2) molecules. STAT 1 and STAT 2 dimerize, translocate into the nucleus and bind to interferon regulatory factor 9 (IRF9) to produce ISG factor 3 (ISGF3) complex associated with IFN‐stimulated response elements. Transcription of ISG is activated, which gives the immune cells antiviral, antibacterial and anti‐apoptotic properties.^30^, ^31^

**FIGURE 3:**
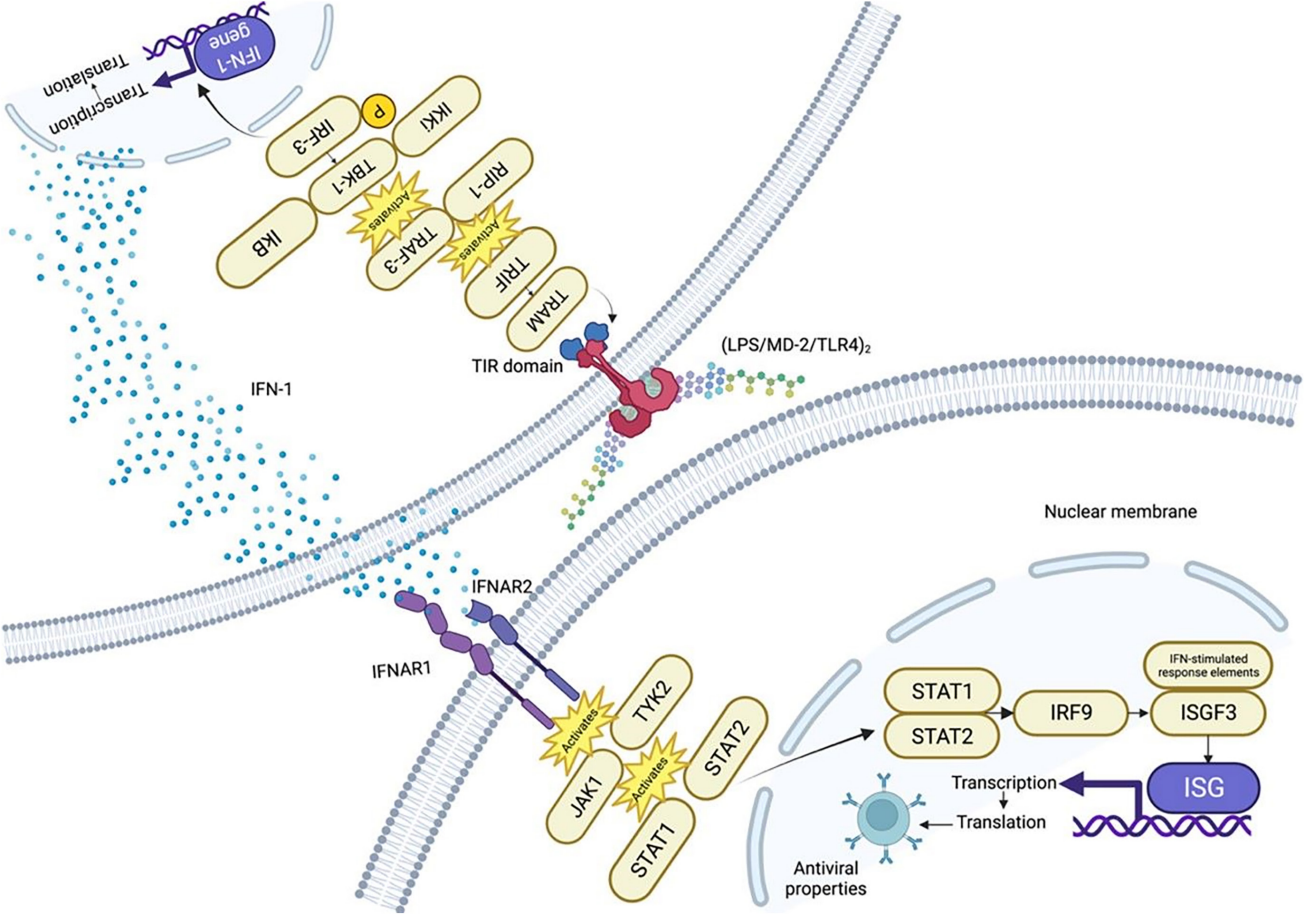
The figure depicts the TRIF‐dependent pathway, outlining molecular events from *TLR4* activation to type‐1 interferon gene transcription. It also illustrates downstream signaling initiated by type‐1 interferon binding to its receptor, featuring key interactions with TRAM, TRIF, RIP‐1, TRAF‐3, TBK‐1, IRF‐3, IFNAR1, IFNAR2, JAK1, TYK2, STAT1, STAT2, and IRF9. IFNAR1, interferon alpha and beta receptor subunit 1; IRF‐3, interferon regulatory factor 3; JAK1, Janus kinase 1; RIP‐1, receptor‐interacting serine/threonine‐protein kinase 1; STAT1, signal transducer and activator of transcription 1; TBK‐1, TANK‐binding kinase 1; TLR 4, toll‐like receptor 4; TRAF‐3, TNF receptor associated factor 3; TRAM, TRIF‐related adaptor molecule; TRIF, TIR‐domain‐containing adapter‐inducing interferon‐β; TYK2, tyrosine kinase 2.

Type‐1 interferon limits the spread of infectious agents, promotes antigen presentation, regulates the function of natural killer cell, stimulates T and B cells responses and induces immunological memory,^23^ as expressed in the Figure 3.

## 5 NF‐KB SIGNAL PATHWAY: ORCHESTRATING THE SYMPHONY OF IMMUNE RESPONSES

NF‐kB is a transcription factor, which regulates a wide range of genes to produce proteins that are involved in immune and inflammatory responses.^32^, ^33^, ^34^ Its nuclear localization is often masked by a family of inhibitory proteins, which includes family members of IkB and precursor proteins. It is unmasked by degradation of these inhibitory proteins through phosphorylation. After nuclear translocation, it binds with the specific DNA component, kB enhancer to form heterodimers or homodimers, initiating the transcription process of the gene.^35^

**FIGURE 4:**
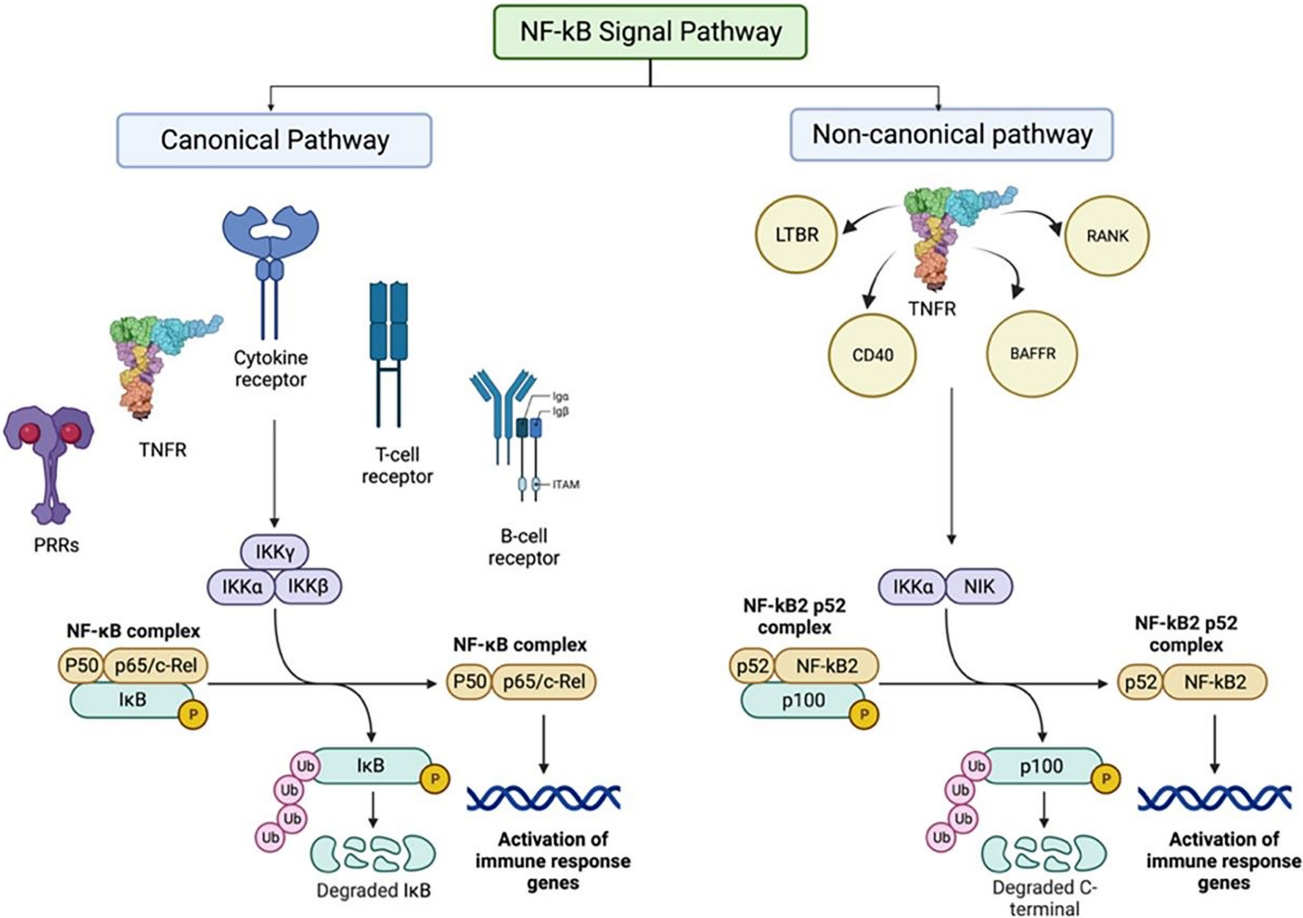
The figure details the nuclear factor kappa light chain enhancer of activated B cells (NF‐kB) signaling pathway, highlighting canonical and noncanonical pathways, their mechanisms, and collaborative role in immune responses. It depicts key interactions, including NF‐kB, inhibitory proteins (IkB, p105, and p100), phosphorylation events, and specific receptors.

There are two different signaling pathways, which are the canonical and noncanonical pathways.^36^ The canonical pathway involves in all aspects of immune responses, and responds to large number of stimuli, including pattern‐recognition receptors (PRRs), TNF receptor (TNFR), cytokine receptors, T‐cell receptor and B‐cell receptor.^37^ The activation of NF‐kB is induced by degradation of IkBα through phosphorylation by the IKK complex at two N‐terminal serines, leading to nuclear translocation of canonical NF‐kB.^32^, ^38^, ^39^, ^40^ In contrast to the canonical NF‐kB pathway, the noncanonical pathway cooperates with the canonical pathway in stimulation of adaptive immune responses, and selectively responds to a specific group of stimuli, including members of TNFR such as lymphotoxin beta receptor (LTBR), cluster of differentiation 40 (CD40), B‐cell activating factor receptor (BAFFR) and receptor activator of nuclear factor kB (RANK)^41^, ^42^ (Figure 4). This pathway involves degradation of C‐terminal IkB‐like structure in NF‐kB2 precursor protein, p100, through phosphorylation by NF‐kB‐inducing kinase (NIK), leading to nuclear translocation of noncanonical NF‐kB.^43^, ^44^

## 6 CYP2E1 SIGNAL PATHWAY: DECODING THE INTRICATE INTERPLAY WITHIN THE CNS

CYP2E1 is a major alcohol‐metabolizing enzyme in the brain that leads to production of ROS and acetaldehyde, which damage the liver and the other internal organs.^45^, ^46^ Results from certain studies suggested the involvement of CYP2E1 in alcohol metabolism in neuron is greater than alcohol dehydrogenase (ADH), as ADH is known to have low level in these cells.^47^ In the brain, CYP2E1 is the only enzyme involved in ethanol oxidation and ROS production, leading to lipid peroxidation, oxidative stress, and apoptosis.^47^, ^48^, ^49^, ^50^ If chronic alcohol consumption is continued, the permeability of the blood–brain barrier increases, and mitochondrial dysfunction causes neurodegeneration through entry of neurotoxic substances such as toxins, pathogens, inflammatory molecules, and harmful proteins.^51^, ^52^, ^53^

**FIGURE 5:**
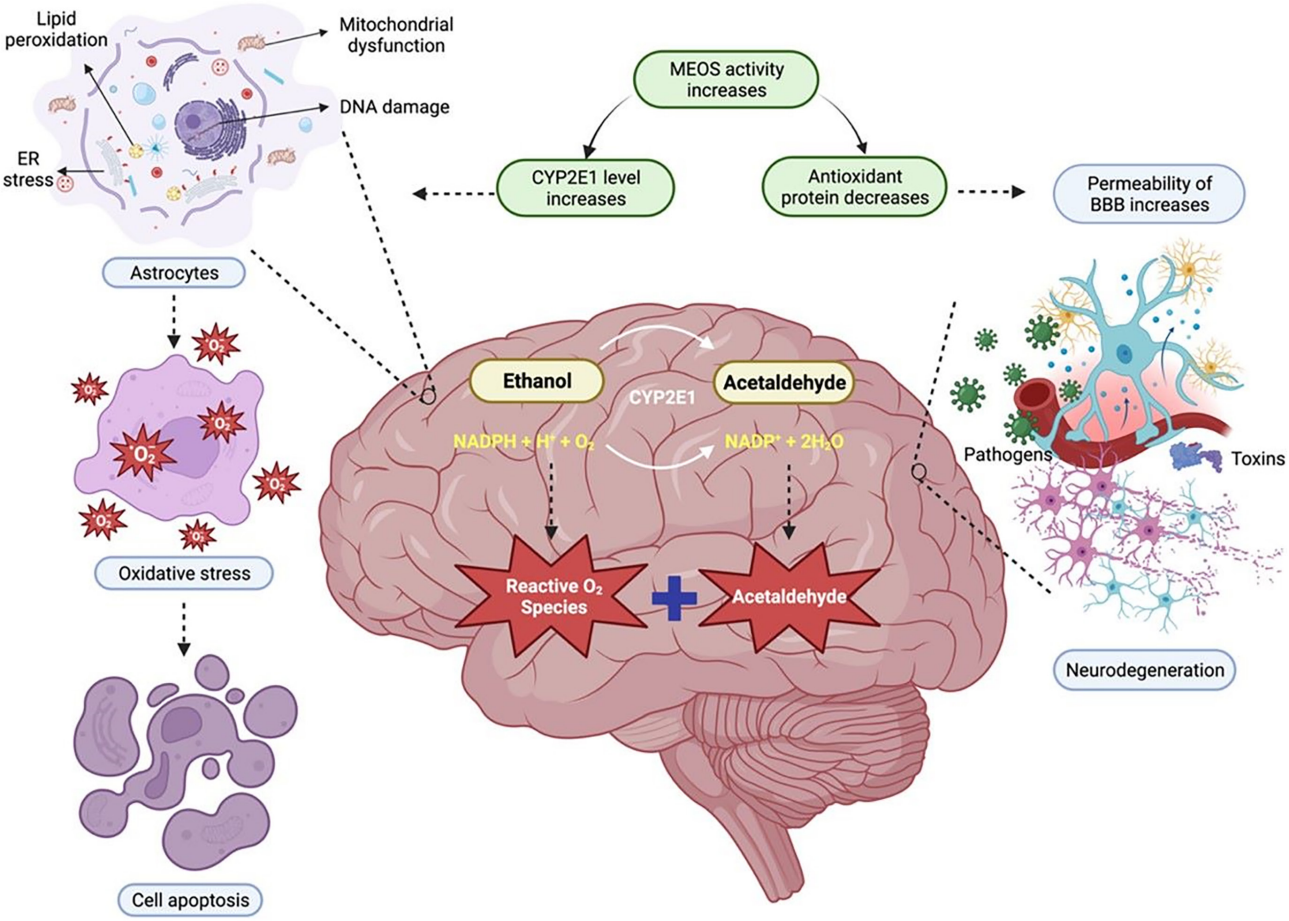
The figure illustrates the Cytochrome P450 2E1 (CYP2E1) signaling pathway in the central nervous system (CNS), emphasizing its role in ethanol metabolism and associated tissue damage risks. It depicts key interactions, including CYP2E1, ethanol metabolism, reactive oxygen species (ROS) production, oxidative stress, and downstream effects on CNS cells.

Some studies have also suggested that CYP2E1 has a major role in microsomal ethanol oxidizing system (MEOS), as it catalyzes the oxidation of ethanol.^49^, ^52^ Chronic alcohol consumption leads to a decrease in the level of antioxidants such as catalase, superoxide dismutase, and glutathione S‐transferase, resulting in cellular damage due to oxidative stress. For instance, an increase in MEOS activity leads to proliferation of smooth endoplasmic reticulum (SER), causing ER stress because there is imbalance between the need for protein folding and the capacity of ER.^52^ To respond to ER stress, a signaling pathway known as the unfolded protein response (UPR) is activated which can trigger cell death if the stress is severe and prolonged.^54^ In addition to that, an increase in CYP2E1 due to high MEOS activity inhibits the function of ADH, resulting in accumulation of acetaldehyde. The combination of high ROS and acetaldehyde damages DNA and protein, reduces oxygen uptake by liver cells and increases glutathione depletion, thereby inhibiting tissue building and repair^52^ (Figure 5).

## 7 ROS PATHWAY: EXPLORING THE IMPACT ON INFLAMMATION AND CELL FATE

**FIGURE 6:**
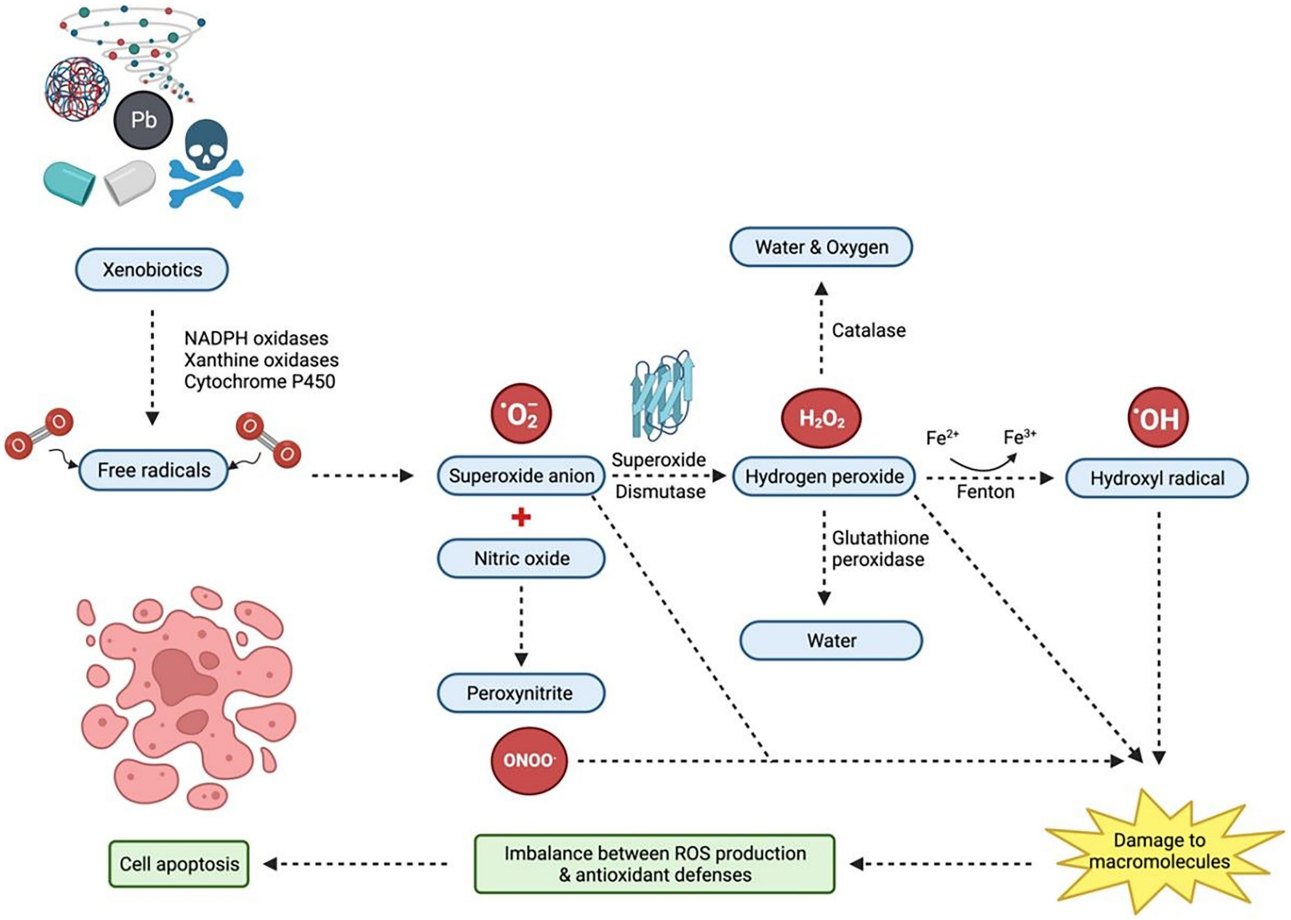
The figure depicts the influence of reactive oxygen species (ROS) pathways on cell fate and inflammation, emphasizing its multifaceted impact on survival, death, differentiation, and anti‐inflammatory factor production. Key interactions involving ROS, free radicals, environmental stressors, production pathways, and downstream effects on macromolecules are shown.

As shown in Figure 6, ROS are small molecules that are temporarily present inside the body and are highly reactive.^55^ They can be oxygen‐derived free radical molecules such as hydroxyl radicals (OH^•^) and superoxide anions (O_2_^•−^) or non‐radical molecules such as hydrogen peroxide (H_2_O_2_).^56^, ^57^ Production of ROS is caused by environmental stress attributed to xenobiotics, which are toxic compounds such as heavy metals, microparticles, nanoparticles, quinone compounds, inflammatory cytokines, environmental toxins, various pharmaceutical agents, UV radiation, ionizing radiation, aldehydes and pesticides that can be catalyzed by NADPH oxidases, xanthine oxidases and cytochrome P450 reductase to produce free radicals.^57^, ^58^, ^59^, ^60^ When these radicals react with oxygen, they will generate superoxide anions (O_2_^•−^) which either react with nitric oxide (NO^•^), a reactive nitrogen species (RNS), to produce peroxynitrite (ONOO^−^), or are catalyzed by superoxide dismutase (SOD) to produce H_2_O_2_.^61^ H_2_O_2_ will either be detoxified by antioxidants such as catalase and glutathione peroxidase to produce water or will undergo the Fenton reaction to produce OH^•^ through reduction. The production of free radical molecules leads to damage to macromolecules such as proteins, lipids, and nucleic acids when there is an imbalance between production of ROS and the antioxidant defenses against ROS, causing oxidative stress and cell apoptosis.^56^, ^60^

## 8 NRF2 PATHWAY: UNVEILING CELLULAR DEFENSE MECHANISMS AGAINST OXIDATIVE STRESS AND INFLAMMATION

Nrf2 is a transcription factor that contributes to anti‐inflammatory and detoxification processes by regulating gene expression to inhibit inflammatory progression due to oxidative damage.^62^ Once the inflammatory response is activated, the immune cells such as lymphocytes, mast cells and monocytes are recruited to the site of injury and generate the production of ROS that damage the macromolecules.^63^, ^64^, ^65^ This process can be inhibited by an anti‐inflammation response by Nrf2.

Nrf2 consists of seven domains, one of which is Nrf2‐ECH homology (Neh)2.^66^ It contains DLG and ETGE, which serve as attachments that interact with Nrf2 and Kelch‐like ECH‐associated protein 1 (Keap1), which inhibits the transcription of Nrf2 to activate antioxidant mechanisms through proteasomal degradation and ubiquitination.^67^, ^68^ Under oxidative stress, Nrf2 dissociates from Keap1 binding due to modification of Keap1.^62^, ^69^ Activated Nrf2 translocates into the nucleus and binds with small musculoaponeurotic fibrosarcoma (Maf) protein, activating the antioxidant response element (ARE) genes.^68^, ^69^, ^70^, ^71^ Nrf2 binds with ARE genes such as heme oxygenase 1 (HO‐1) to deactivate the inflammatory response.^72^ HO‐1 breaks down heme into carbon monoxide (CO) and free ions and breaks down biliverdin into bilirubin.^65^, ^73^ Degradation of pro‐inflammatory free heme leads to reduced production of proinflammatory cytokines. CO acts as an anti‐inflammatory compound that inhibits the NF‐kB signaling pathway, while bilirubin acts as a strong antioxidant that protects the cells from oxidative stress, and suppresses hepatitis and autoimmune encephalomyelitis, a condition where the body's immune system attacks the brain.^73^

**FIGURE 7:**
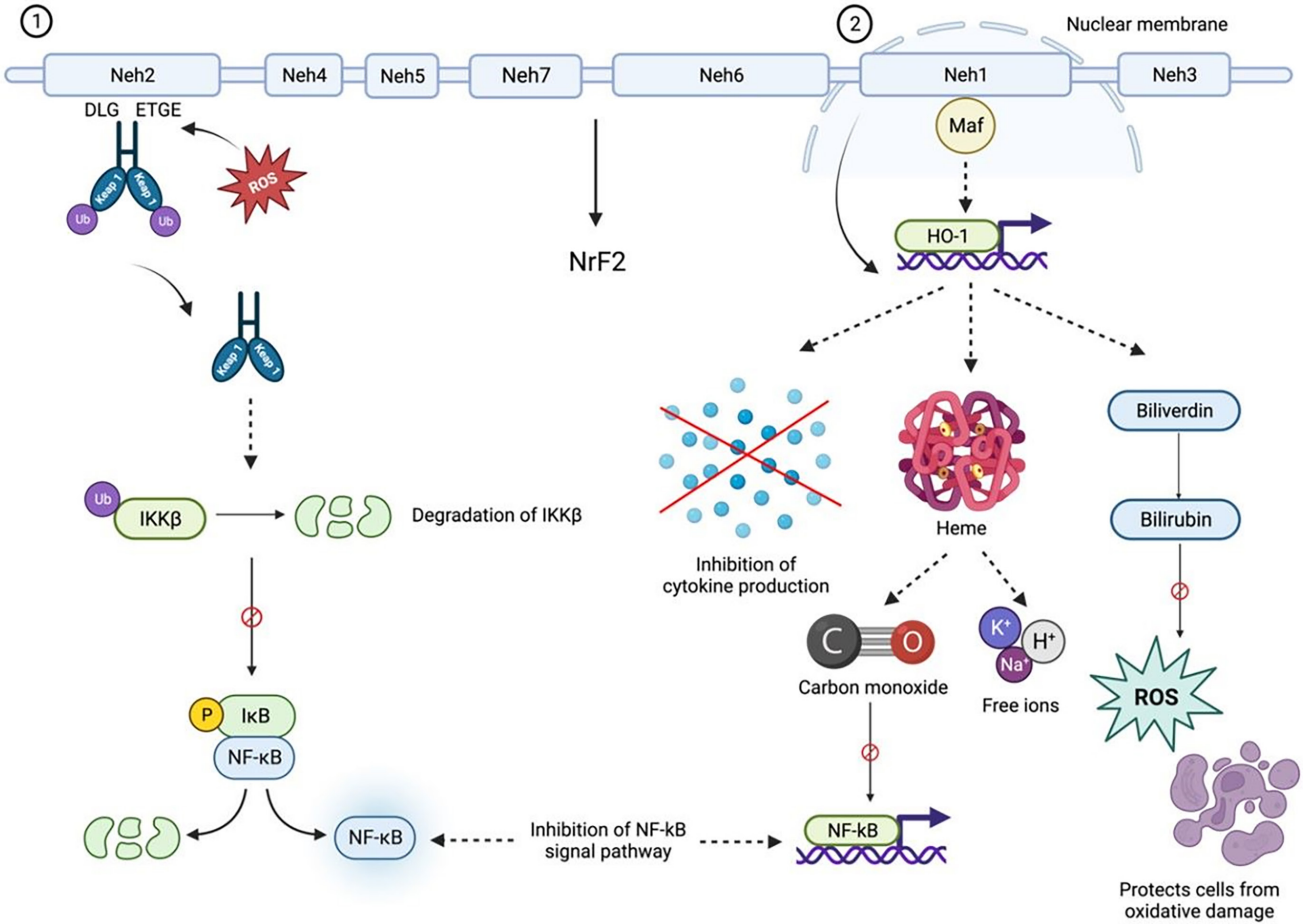
The figure depicts the role of the nuclear factor erythroid 2‐related factor 2 (Nrf2) pathway in anti‐inflammation and defense against oxidative stress. It shows key interactions, including Nrf2, Keap1, and ARE gene activation, and downstream effects on inflammatory mediators and cell adhesion molecules.

There are three ways for Keap1/Nrf2/ARE signaling pathway to inhibit generation of NF‐kB, thereby inhibiting the production of proinflammatory cytokines. First, Keap1 degrades IKKβ through ubiquitination and proteosome degradation, inhibiting the phosphorylation of inhibitory proteins and preventing NF‐kB from translocating into the nucleus for gene transcription.^74^ Second, oxidative stress activates the transcription of NF‐kB to produce proinflammatory cytokines such as cyclooxygenase‐2 (COX‐2). The terminal product of COX‐2, 15‐deoxy‐Δ^12^, ^14^‐prostaglandin J_2_ (15d‐PGJ2), induces the Nrf2 pathway.^75^ Third, Nrf2 binds with CREB‐binding protein (CBP), small Maf and other transcriptional cofactors to drive the transcription of ARE genes.^76^ Both activation and inhibition occur between NF‐kB pathway and Nrf2 pathway. Increased expression of Nrf2 inhibits NF‐kB transcriptional activity while decreased expression of Nrf2 increases NF‐kB transcriptional activity, showing that there is balance between inflammatory and anti‐inflammatory responses^72^, ^77^, ^78^ (Figure 7).

## 9 ALCOHOL'S MULTI‐ORGAN IMPACT: UNDERSTANDING THE COMPLEXITIES OF INTERNAL ORGAN DAMAGE

After alcohol is ingested, it is absorbed from the small intestine into the veins that collect blood from the stomach and bowels and is then carried to the liver via the portal vein. Alcohol readily diffuses across membranes and distributes through all cells and tissues and affects cell function by producing harmful byproducts and interacting with certain protein and cell membranes, stimulating the immune signaling pathway.^79^, ^80^ At high concentrations or during chronic alcohol consumption, alcohol is eliminated at a high rate and metabolizing enzymes are overproduced because of high enzymatic activity.^81^

Alcohol is metabolized by two different pathways, namely oxidative and non‐oxidative pathways. In the oxidative pathway, ADH metabolizes alcohol and generates acetaldehyde, which forms ROS after it reacts directly with oxygen.^82^ This reaction involves the reduction of ^+^nicotinamide adenine dinucleotide (NAD^+^) by two electrons, forming nicotinamide adenine dinucleotide + hydrogen (NADH). This process mainly occurs in the liver as ADH is located in the cytosol of liver cells.^83^ Acetaldehyde, ADH, ROS, and NADH produced from this reaction are harmful to body tissues and organs. Acetaldehyde interacts with certain proteins such as lipoproteins, tubulin, hemoglobin, albumin, collagen, and cytochrome enzymes to form adducts, which will be recognized as foreign substance and attacked by immune cells, causing liver inflammation. ADH forms adducts with dopamine in the brain to form salsolinol, which may cause alcohol dependence, and with DNA to form carcinogenic DNA adducts, producing cancerous cells, and developing alcohol‐associated tumors.^84^ ADH also binds to proteins such as microtubules, microsomal proteins, and enzymes to form protein adducts, which impair protein secretion, causing enlargement of liver, known as hepatomegaly.^85^, ^86^ ROS activate the body defense mechanism and stimulate the release of inflammatory cytokines, such as tumor necrosis factor alpha (TNF‐α), that can contribute to scar tissue formation in the liver due to inflammation, known as fibrosis.^87^ Furthermore, ROS combine with DNA, lipids and proteins, and alter the membrane permeability of mitochondria through peroxidation, causing molecules contained in the mitochondria, such as cytochrome c, to be released into the cytosol, inducing a cascade of biochemical reactions that cause cell death.^88^, ^89^ ROS also alter the distribution of electrical charges across the mitochondrial membrane, reducing the level of adenosine triphosphate (ATP), and disrupting the cellular respiration process that stores energy for cell survival. The production of NADH from this reaction increases the NADH: NAD^+^ ratio, resulting in more electrons passing through the electron transport chain, and attaching to free oxygen to form superoxide radicals, causing tissue damage.^90^, ^91^, ^92^, ^93^

Two pathways of alcohol metabolism occur in non‐liver tissues such as brain, heart, and lungs that do not contain ADH or contain only low levels ADH. This type of metabolism is catalyzed by CYP2E1 and catalase.^94^ CYP2E1 is an enzyme present in the microsomes that metabolizes alcohol into acetaldehyde when there is high rate of consumption of alcohol.^95^ CYP2E1 disrupts the CYP2E1‐mediated metabolism of medication such as pain killers, blood thinners, propranolol, and the sedative diazepam, as during chronic alcohol consumption more of this enzyme is used to metabolize alcohol, causing a reduction in the efficacy of the medication. Moreover, CYP2E1 activates the pro‐carcinogens found in tobacco smoke, increasing the risk of getting cancer in the esophagus, oral cavity, and larynx.^96^, ^97^

**FIGURE 8:**
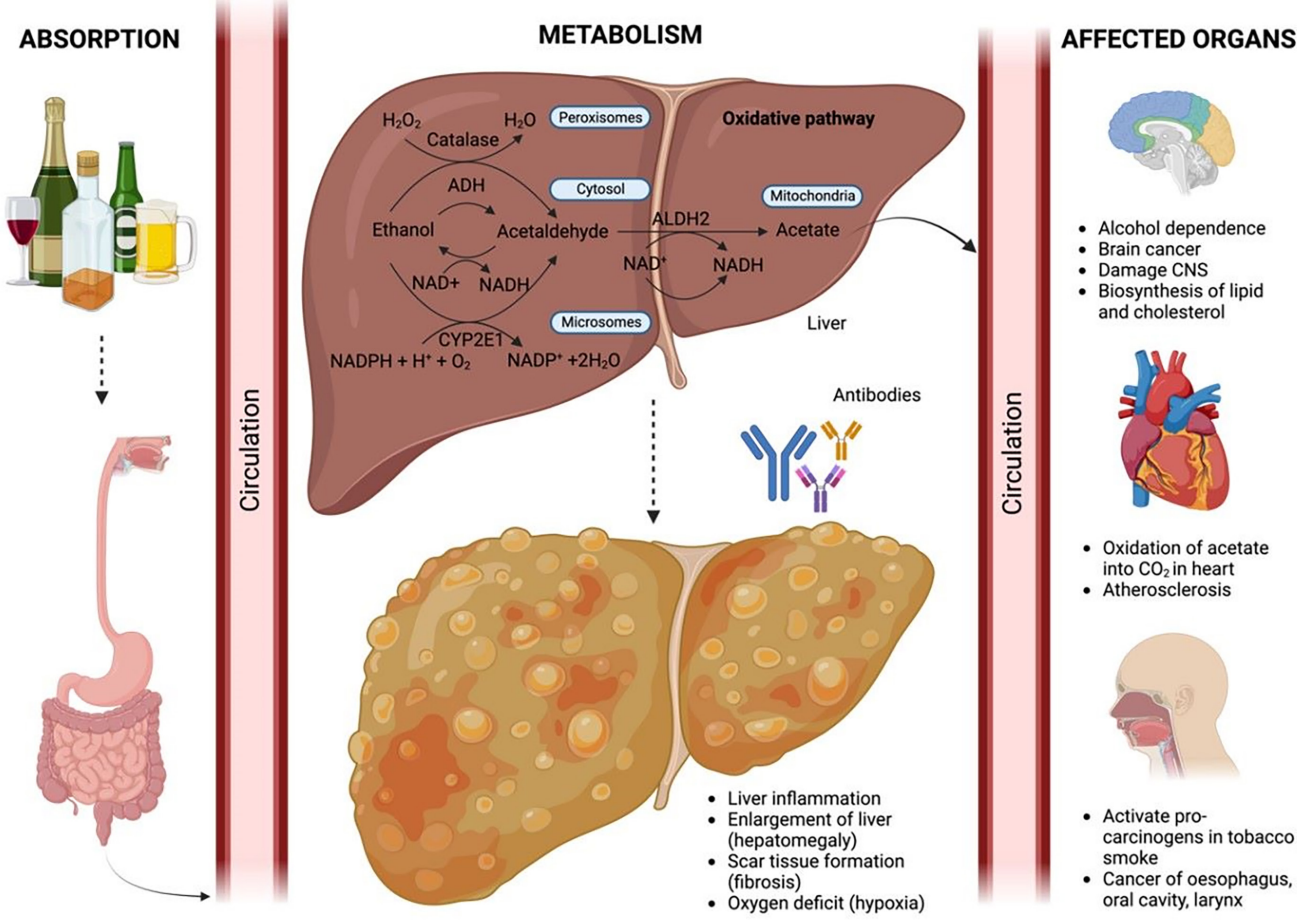
The figure shows alcohol's impact on organs, focusing on absorption, metabolism, and effects on various systems. Key interactions include alcohol metabolism, liver damage, oxidative stress, and downstream effects on tissues.

After oxidation of alcohol into acetaldehyde has occurred, acetaldehyde is further oxidized into acetate by aldehyde dehydrogenase 2 (ALDH2), and NAD^+^ is simultaneously reduced to NADH. This process primarily occurs in liver, so any damage caused associated with alcohol metabolism by these enzymes would affect that organ.^98^, ^99^ The production of acetate and NADH by this reaction leads to adverse effects in different organs. Acetate escapes from the liver into the blood and is further oxidized into carbon dioxide (CO_2_) in heart, skeletal muscle, and brain, damaging the CNS and other metabolic processes occuring in the affected organs.^85^ Acetate is also metabolized to acetyl coenzyme A (acetylCoA) which is involved in the biosynthesis of lipid and cholesterol in mitochondria of peripheral and brain tissues.^100^ The production of NADH has the potential to cause liver cell damage due to oxygen deficit in these cells, known as hypoxia. This is because NADH is oxidized by a series of chemical reactions in mitochondria that transfer electrons to oxygen (O_2_), forming oxide ions (O^2−^). This reaction increases the demand for oxygen because more oxygen is needed to accept the electrons, causing a lack of oxygen to carry out cellular respiration, and eventually leading to cell death,^101^, ^102^ as shown in Figure 8.

## 10 NONOXIDATIVE PATHWAY: UNRAVELING THE VARIED MANIFESTATIONS OF ALCOHOL‐INDUCED DAMAGE

Besides the oxidative pathway, a smaller fraction of alcohol is metabolized through a non‐oxidative pathway to produce metabolites such as ethyl glucuronide (EtG), ethyl sulphate (EtS), phosphatidylethanol (PEth) and fatty acid ethyl ester (FAEE) that will cause tissue damage. These are the biomarkers of alcohol consumption, and they will be detected in the blood, urine or hair of heavy drinkers over a longer period than in light drinkers.^103^, ^104^ Although a relatively small portion of alcohol is metabolized through this pathway, the damaging effects of will be prolonged due to the slower elimination of the metabolites through excretion; the metabolites will remain in the body fluids longer than alcohol.^105^

EtG is formed when the enzyme glucuronyl moiety from uridine 5′‐diphospho (UDP)‐ glucuronic acid is transferred to ethanol, catalyzed by UDP‐ glucuronosyltransferases (UGTs), while Ets is formed by sulphonation of ethanol, which is catalyzed by sulphotransferases (SULT).^106^ EtG has been shown to activate *TLR4* signaling, causing allodynia, which is the condition where a stimulus that normally does not cause pain causes a feeling of pain.^107^

PEth is formed by transphosphotidylation of phospholipids with ethanol, catalyzed by phospholipase D (PLD).^82^, ^105^ If there is a high concentration of alcohol, PEth synthesis disrupts other PLD‐mediated cellular processes such as synthesis of phosphatidic acid (PA), as PLD is needed in those processes.^108^ Studies have shown that formation of PEth promotes intestinal hyperplasia, which is the change in the mucous membrane of stomach and intestinal epithelium associated with cancer development, and influences other phospholipid signaling pathways that affect structural properties, and the function and fluidity of biomembranes.^105^, ^109^

**FIGURE 9:**
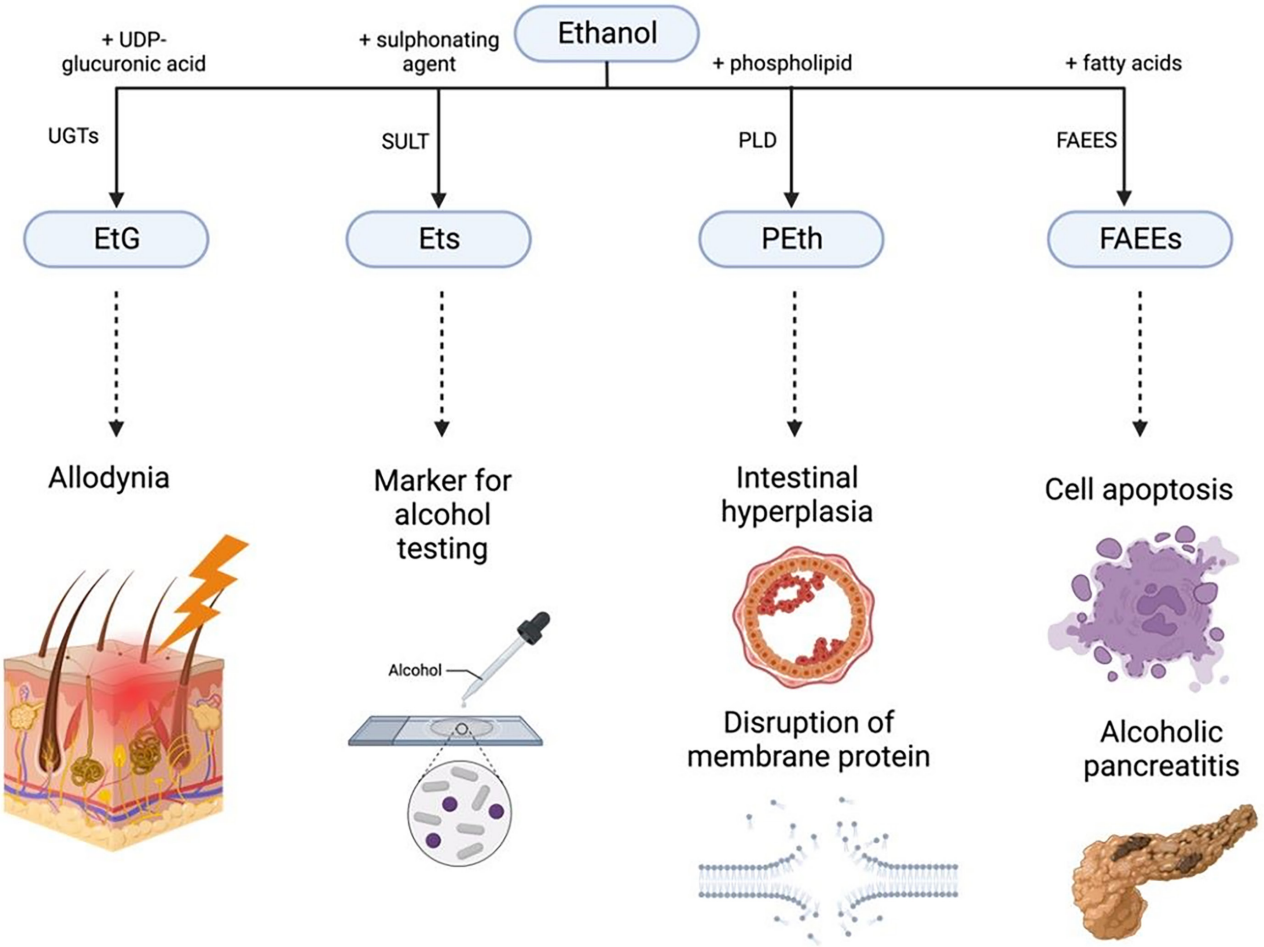
Nonoxidative pathway‐mediated alcohol damage. The figure illustrates the diverse effects of alcohol‐induced tissue damage, highlighting the biomarker metabolites (EtG, EtS, PEth, FAEE) and their impact on cellular signaling and organelle functions.

FAEEs are formed through the enzymatic esterification of ethanol with fatty acids, catalyzed by FAEE synthase (FAEES). FAEEs cause ethanol‐induced toxicity, which leads to inhibition of cell proliferation, destabilization of lysosomes, mitochondrial depolarization, and induction of apoptosis.^110^, ^111^ The combination of ethanol and fatty acids also causes inflammation, necrosis, alcoholic pancreatitis, mitochondrial depolarization, depletion of cellular ATP and sustained elevations of intracellular Ca^2+^ ^112^ (Figure 9).

## 11 IMPACTS OF ALCOHOL ON REPRODUCTIVE HEALTH: UNVEILING GENDER‐SPECIFIC RAMIFICATIONS

### 11.1 Effects of alcohol on female reproductive health

Acute alcohol consumption in females significantly increases oestradiol, causing alterations in oestrous cycling.^113^ The increase in testosterone caused by chronic alcohol consumption suppresses the HPG unit.^114^ Some studies have shown that the effect includes a reduction in the level of estrogen, which alters maturation of follicles. Low estrogen levels decrease the secretion of luteinizing hormone‐releasing hormone (LHRH) from the hypothalamus, causing a decrease in luteinizing hormone (LH) production, and inhibiting ovulation.^115^ Besides its effect on HPG activity, chronic alcohol consumption can also affect female reproduction via the opioid pathway. Research has shown that alcohol increases opioid activity in the brain, inhibiting the secretion of hypothalamic LHRH and inhibiting the HPG axis.^116^, ^117^ Alcohol also lowers the level of insulin‐like growth factor (IGF‐1) in the bloodstream, which has the function of activating LHRH release and increasing the production of LH.^118^, ^119^ All these disruptions in hormone levels in the reproductive system cause irregular menstrual cycles, inhibition of ovulation, infertility, and early menopause. Besides suppressing ovulation, heavy alcohol consumption can affect ovarian reserves, which are a measure of a woman's reproductive potential, based on the number of oocytes available in the ovary during the woman's lifetime. This can be determined by measuring the levels of follicle stimulating hormone (FSH) and anti‐Mullerian hormone (AMH), with low levels indicating a lower reproductive potential due to binge drinking alcohol.^120^, ^121^, ^122^

### 11.2 Effects of alcohol on male reproductive health

**FIGURE 10:**
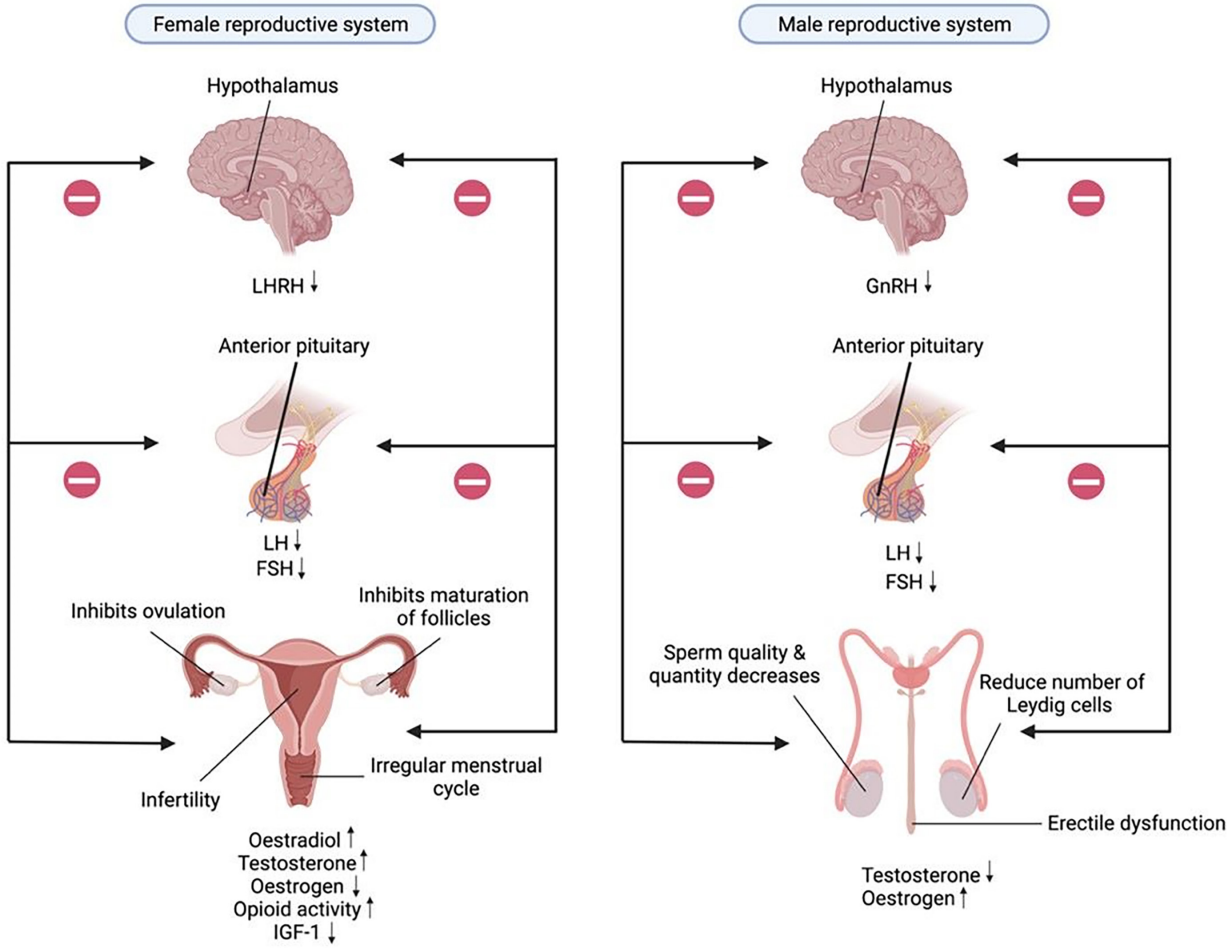
Alcohol's impact on reproductive health. This figure explores the gender‐specific effects of alcohol on reproductive health, highlighting disruptions in hormone levels. In females, alcohol alters oestradiol and testosterone, affecting the HPG unit and opioid pathway, leading to menstrual irregularities and infertility. In males, chronic alcohol consumption disrupts the hypothalamic‐pituitary‐gonadal (HPT) axis, reducing GnRH and gonadotropin levels, resulting in decreased testosterone production and reproductive dysfunctions.

Chronic alcohol consumption in males disrupts the hypothalamic–pituitary‐testicular (HPT) axis, affecting the male reproductive glands by significantly reducing the level of gonadotropin releasing hormone (GnRH) due to the disruption of the nitric oxide (NO) pathway. Excessive alcohol consumption prevents NO from binding to the heme group of cyclooxygenase‐1, which helps in the synthesis of GnRH, because alcohol inhibits cyclooxygenase activity.^9^ As a result, the levels of LH and FSH will be reduced as GnRH triggers their production in the pituitary gland, causing a reduction in testosterone levels and numbers of Leydig cells.^123^ Low levels of FSH and LH will also cause loss of secondary sexual characteristics such as erectile dysfunction, issues with arousal and desire, infertility, hypogonadism, reduced sperm production, and impotence.^9^ Besides inhibition of the HPT axis, high alcohol consumption also reduces testosterone levels by stimulating the activity of aromatase which converts testosterone to oestradiol. When the estrogen level is elevated, the synthesis of FSH and GnRH is inhibited, which results in low sperm production.^124^, ^125^ In addition, production of ROS from alcohol metabolism causes protein and DNA damage in the sperm cells, increased risk of cell apoptosis, abnormalities in meiotic division, reduced gamete viability, poorly condensed chromatin, and disruption in sperm maturation due to changes in gene regulation, changes in mitochondrial ribonucleic acid (RNA), and accelerated loss of the acrosome due to oxidative damage of membrane lipids and proteins that alters the membrane's permeability and lipid fluidity. This makes it difficult for the sperms to penetrate the egg's coat, reducing fertilizing rates. Furthermore, the production of ROS causes oxidative damage of the epididymis due to loss of antimicrobial properties as a result of altered mRNA expression of β‐defensin, and disruption in expression of proliferating cell nuclear antigen (PCNA) in germ cells, resulting in cell apoptosis, and reduced production of sperm cells,^125^, ^126^, ^127^ as shown in Figure 10.

Alcohol metabolism via Cytochrome P450 2E1 (CYP2E1) generates ROS, leading to oxidative stress in reproductive organs. This oxidative stress can disrupt cellular function, damage DNA, and impair reproductive hormone regulation, ultimately affecting fertility and reproductive health. Activation of the Nrf2 pathway serves as a protective mechanism against oxidative damage, but chronic alcohol consumption may overwhelm this defense mechanism, exacerbating reproductive dysfunction. Understanding these pathways provides insight into how alcohol impacts reproductive health at the molecular level, facilitating the development of targeted interventions to mitigate alcohol‐related reproductive issues.

### 11.3 Gender differences in alcohol‐induced damage

Previous studies have shown that negative psychological effects due to problematic drinking are more significant in women than men, as there is gender difference in alcohol pharmacokinetics and neurotransmitter systems and the influence on these of gonadal steroid hormones. In terms of pharmacokinetics, women have a higher blood alcohol concentration than men despite consuming the same amount of alcohol because women have a lower proportion of body water than men, and ADH activity in stomach and liver in women is lower than in men due to a difference in gastric mucosa ADH activity. Thus, an alcohol molecule in women takes longer to be metabolized, resulting in a more persistent high alcohol blood level in women compared to men after consumption of alcohol. Regarding neurotransmitters, there is a difference in neurotransmitter release and neurotransmitter receptor subunit expression between men and women in response to alcohol consumption. In addition, the effect of alcohol consumption on gonadal hormones in women is greater, and many neurobiological responses are affected by changes in estrogen levels, and the concentration of serotonin and serotonin‐receptor subtypes, increasing the risk of depression or mental health issues in women after chronic alcohol consumption.^128^ Therefore, it is important for healthcare practitioners and policymakers to work together to mitigate alcohol‐induced damage and alcohol abuse through various clinical interventions such as screening, brief interventions, and referral for treatment (SBIRT). In terms of screening, the U.S. Preventive Task Force recommends the Alcohol Use Disorders Identification Test (AUDIT) to assess the frequency of alcohol consumption. Brief interventions can range from brief written or verbal advice to motivational interviews with trained advisers offering suggestions on the patient's behavior in relation to alcohol consumption. Referral for treatment will be more effective among populations with severe alcohol‐related problems as it involves more intensive interventions.^129^

## 12 CONCLUSION

In conclusion, TLR/NF‐kB plays a pivotal role in inflammatory and immune responses to excessive alcohol consumption, increasing the likelihood of bacterial endotoxins invading liver cells due to chronic alcohol consumption. On the other hand, CYP2E1/ROS/Nrf2 is a key pathway in oxidative stress and antioxidant responses. The excessive production of liver enzymes and the generation of ROS, NADH, acetaldehyde, and acetate can lead to damage in internal organs such as the heart, brain, and lungs. Furthermore, excessive alcohol intake adversely affects reproductive health by inhibiting the HPG unit, resulting in infertility.

## AUTHOR CONTRIBUTIONS

E.K.Q.Z and V.S. conceived and designed the structure of the review. E.K.Q.Z conducted literature research and drafted the entire manuscript. V.S. and N.S.A.L edited the manuscript. E.K.Q.Z and V.S. contributed to the key parts of the text associated with it. All authors have read and agreed to the published version of the manuscript.

## FUNDING INFORMATION

There is no funding support for this review study. The entire study was supported by the Jeffrey Cheah School of Medicine and Health Sciences and the Library Resources, Monash University Malaysia.

## CONFLICT OF INTEREST STATEMENT

The authors declare no conflict of interest.

## ETHICS STATEMENT

Ethical approval was not required for this review study as it did not involve primary data collection or human or animal subjects. The entire study was supported by the Jeffrey Cheah School of Medicine and Health Sciences, Monash University Malaysia.
